# Outcomes of patients with advanced cancer and *KRAS* mutations in phase I clinical trials

**DOI:** 10.18632/oncotarget.2339

**Published:** 2014-08-10

**Authors:** Rabih Said, Yang Ye, Gerald Steven Falchook, Filip Janku, Aung Naing, Ralph Zinner, George R. Blumenschein, Siqing Fu, David S. Hong, Sarina Anne Piha-Paul, Jennifer J. Wheler, Razelle Kurzrock, Gary A. Palmer, Kenneth Aldape, Kenneth R. Hess, Apostolia Maria Tsimberidou

**Affiliations:** ^1^ Department of Investigational Cancer Therapeutics, The University of Texas MD Anderson Cancer Center, Houston, TX; ^2^ Department of Internal Medicine, The University of Texas Health Science Center at Houston, Houston, TX; ^3^ Department of Thoracic Head and Neck Medical Oncology, The University of Texas MD Anderson Cancer Center, Houston, TX; ^4^ Department of Internal Medicine, Moores Cancer Center - University of California San Diego, LaJolla, CA; ^5^ Foundation Medicine, Inc., Cambridge, MA; ^6^ Department of Molecular Pathology, The University of Texas MD Anderson Cancer Center, Houston, TX; ^7^ Department of Biostatistics, The University of Texas MD Anderson Cancer Center, Houston, TX

**Keywords:** Personalized medicine, Phase I, Clinical trials, Targeted therapy, Molecular alterations

## Abstract

**Background:**

*KRAS* mutation is common in human cancer. We assessed the clinical factors, including type of *KRAS* mutation and treatment, of patients with advanced cancer and tumor *KRAS* mutations and their association with treatment outcomes.

**Methods:**

Patients referred to the Phase I Clinic for treatment who underwent testing for *KRAS* mutations were analyzed.

**Results:**

Of 1,781 patients, 365 (21%) had a *KRAS* mutation. The G12D mutation was the most common mutation (29%). *PIK3CA* mutations were found in 24% and 10% of patients with and without KRAS mutations (p<0.0001). Of 223 patients with a *KRAS* mutation who were evaluable for response, 56 were treated with a MEK inhibitor-containing therapy and 167 with other therapies. The clinical benefit (partial response and stable disease lasting ≥ 6 months) rates were 23% and 9%, respectively, for the MEK inhibitor versus other therapies (p=0.005). The median progression-free survival (PFS) was 3.3 and 2.2 months, respectively (p=0.09). The respective median overall survival was 8.4 and 7.0 months (p=0.38). Of 66 patients with a KRAS mutation and additional alterations, higher rates of clinical benefit (p=0.04), PFS (p=0.045), and overall survival (p=0.02) were noted in patients treated with MEK inhibitor-containing therapy (n=9) compared to those treated with targeted therapy matched to the additional alterations (n=24) or other therapy (n=33).

**Conclusions:**

MEK inhibitors in patients with *KRAS-*mutated advanced cancer were associated with higher clinical benefit rates compared to other therapies. Therapeutic strategies that include MEK inhibitors or novel agents combined with other targeted therapies or chemotherapy need further investigation.

## INTRODUCTION

The KRAS gene, a Kirsten ras oncogene homolog from the mammalian ras gene family[1], encodes a protein that is a member of the small GTPase superfamily[2]. The KRAS protein exists in an active form (KRAS-ATP) and in an inactive form (KRAS-ADP), both of which are tightly controlled by the guanine nucleotide exchange factors (GEFs) and GTPase-activating proteins (GAPs)[2-4]. KRAS is a key intracellular protein that activates multiple pathways, including the MAPK and PI3K pathways[5].

Mutant KRAS proteins are GAP-insensitive; therefore, the protein is constitutively GTP-bound, which leads to persistent and independent activation of the downstream effectors[4]. In addition, certain tumor types are associated with specific KRAS mutations, which differ in their carcinogenic potential[6-10]. The high frequency (up to 30%) of RAS mutation in human cancers[11] has stimulated the development of targeted agents against KRAS. Farnesyltransferase inhibitors, which were the first agents used to block the binding of RAS isoforms to the cancer cell membrane, had disappointing results[12-14]. In recent years, the focus has shifted to inhibiting the downstream signaling pathways of RAS. Clinical trials with MEK inhibitors as single agents and/or in combination with cytotoxic agents have been completed[15-19].

Mutations in the KRAS gene, which occur commonly in codons 12, 13, and 61, encode for a single amino-acid substitution. Emerging data demonstrate that each amino-acid substitution may affect the activity of KRAS differently, leading to a different affinity for the various downstream molecules[6]. For example, the G12D mutation encodes for a protein with high affinity for PI3K, while the G12C and G12V mutations activate RAS-like GTPase.

The clinical outcomes of patients with advanced cancer and KRAS mutations by type of codon and amino-acid substitution have not been systematically explored. Therefore, we retrospectively reviewed data from patients with advanced cancer and KRAS mutations who were treated in the Phase I Clinical Trials Program at The University of Texas MD Anderson Cancer Center. Our objectives were to assess the clinical factors, including type of KRAS mutation and treatment, of patients with tumor KRAS mutations and their association with treatment outcomes.

## RESULTS

### Patient characteristics

**Figure 1:**
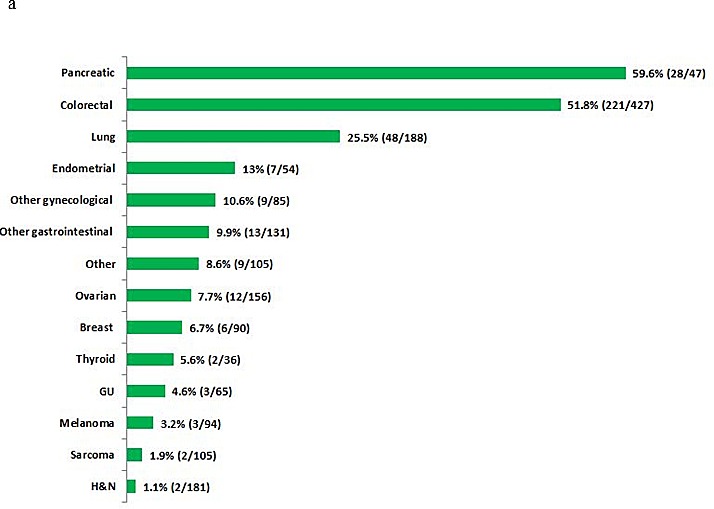

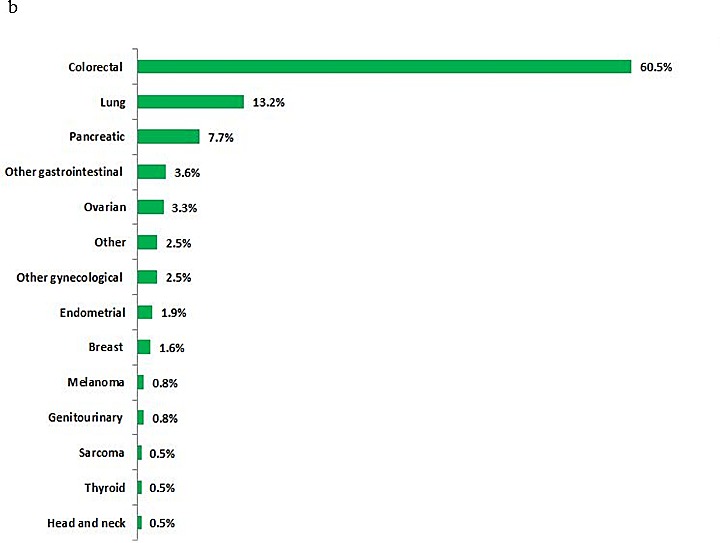
**a.** Proportion of *KRAS* mutations by tumor type in patients tested for *KRAS* mutations. **b.** Distribution of tumor types in patients with *KRAS* mutations (n=365)

Of the 1,781 patients, 365 (20.5%) were found to have a *KRAS* mutation. The clinical and demographic characteristics and tumor types of the patients with and without a *KRAS* mutation are shown in Table 1. Briefly, there was no difference in age (median, 59; p=0.62) or sex (male, 47.3% vs. 46.3%, respectively; p= 0.71) between the patients with wild-type and mutated *KRAS*. However, there was a significant difference in the percentage of patients with *KRAS*-mutated disease by race (African American 29%, Asian 22%, White 20%; p=0.03). The occurrence of *KRAS* mutations varied by tumor type, as summarized in Table 1 and Figure 1a. The most commonly analyzed tumor types were colorectal, lung, and ovarian cancer, reflecting the pattern of referrals to the Phase I Clinic (Figure 1b). Molecular analyses were performed on tumor samples from the primary site in 198 (54%) patients and from a metastatic site in 167 (46%) patients.

**Table 1:** Baseline characteristics of patients with advanced cancer (any tumor type) who were tested for a KRAS mutation

	Total Patients, N=1781	Wild-type *KRAS*, N=1416 (%)	Mutant *KRAS*, N=365 (%)	*p*
Age at Phase I Program presentation (median, range)	59 (4-90)	59 (4-90)	59 (20-84)	0.62
Sex				0.71
Male	840	671 (80)	169 (20)	
Female	941	745 (79)	196 (21)	
Race				0.03
White	1339	1079 (80)	260 (20)	
African American	174	124 (71)	50 (29)	
Hispanic	162	127 (78)	35 (22)	
Asian	6	5 (83)	1 (17)	
Other	100	81 (81)	19 (19)	
Type of cancer				NA
Colorectal	427	206 (48)	221 (52)	
Lung	188	140 (74)	48 (26)	
Pancreatic	47	19 (40)	28 (60)	
Endometrial	54	47 (87)	7 (13)	
Ovarian	156	144 (92)	12 (8)	
Other GI^*^	131	118 (90)	13 (10)	
Other GYN^¤^	85	76 (89)	9 (11)	
Thyroid	36	34 (94)	2 (6)	
Other	657	632(96)	25(4)	
Number of metastatic sites				
>2	518	386 (75)	132 (25)	0.0008
Number of prior therapies				
Median (range)	4 (0-15)	4 (0-15)	3 (0-12)	0.53

* Gastrointestinal

¤ Gynecological

Patients with *KRAS* mutations were more likely to have more than two metastatic sites than patients with wild-type *KRAS* (36% vs. 27%, respectively; p=0.0008). No difference in the number of prior therapies was noted between patients with wild-type and mutated *KRAS* (p=0.53).

### Specific KRAS mutations and other alterations

The distribution of specific *KRAS* mutations by tumor type is summarized in Table 2. Overall, mutations in codons 12 and 13 were the most common. The G12D mutation was the most common mutation overall (29%) in all tumor types except for lung cancer, in which G12C and G12V were more frequent. No G12C or codon 13 (G13D, G13C, G13R, and G13V) mutations were noted in the 28 patients with pancreatic cancer.

**Table 2:** Distribution of *KRAS* mutations by tumor type

*KRAS* Mutation	CRC^*^ (%)	Lung (%)	Pancreatic (%)	Other (%)	Total
G12D	68 (31)	8 (17)	12 (43)	18 (26)	106 (29)
G12V	51 (23)	10 (21)	6 (21)	13 (19)	80 (22)
G12C	13 (6)	19 (40)	0	9 (13)	41 (11)
G13D	29 (13)	2 (4)	0	9 (13)	40 (11)
G12A	15 (7)	4 (8)	0	6 (9)	25 (7)
G12S	14 (7)	1 (2)	0	0	15 (4)
Q61H	6 (3)	2 (4)	1 (4)	5 (7)	14 (4)
G13C	2 (1)	1 (2)	0	1 (1)	4 (1)
Other	16 (7)	0	8 (29)	7 (10)	40 (11)
Unknown	7(3)	1 (2)	1 (4)	0	9 (2)
Total	221	48	28	68	365

* Colorectal cancer

Among the 365 patients with *KRAS* mutations, 256 (70%) were found to have tumors harboring *KRAS* mutations without any other molecular alterations. Alternatively, 109 (30%) patients were found to have tumors with ≥1 additional molecular alterations. The distribution of the various additional molecular alterations in both mutated and wild-type *KRAS* is summarized in Table 3. The number of additional molecular alterations was higher in mutated *KRAS* than in wild-type *KRAS*. The most frequently tested alterations were *PI3KCA*, *BRAF*, and *EGFR*; the most common alterations found in both wild-type and mutant *KRAS* tumors were *PI3KCA*, *p53*, and PTEN. A higher percentage of patients with mutated *KRAS* had *PIK3CA* mutations compared to patients with wild-type *KRAS* (24% vs. 10%, respectively; *p*<0.0001). In contrast, more patients with wild-type *KRAS* had *BRAF* mutations compared with patients with mutated *KRAS* (7% vs. 0.4% respectively; p=0.0002).

**Table 3:** Distribution of other molecular alterations by KRAS mutational status

	Total, n= 1781	Wild-type *KRAS*, n= 1416	Mutant *KRAS*, n= 365	P
No. of associated alterations				
Median (range)	0 (0-9)	0(0-9)	1(1-9)	<0.0001
>2	118	81(6%)	37 (10%)	0.0025
Type of associated alterations				
PI3KCA	162/1386 (12%)	118/1203 (10%)	44/183 (24%)	<0.0001
P53	184/510 (36%)	155/427 (36%)	29/83 (35%)	0.87
PTEN	132/1040 (13%)	111/881 (13%)	21/159 (13%)	0.83
BRAF	77/1370 (6%)	76/1139 (7%)	1/231 (0.4%)	0.0002
EGFR	54/1205 (5%)	49/1022 (5%)	5/183 (3%)*	0.21
MET	41/951(4%)	32/786 (4%)	9/165 (5.5%)	0.43

* Four patients had an EGFR mutation and one patient had EGFR overexpression

### Outcomes of patients with KRAS mutations

**Figure 2:**
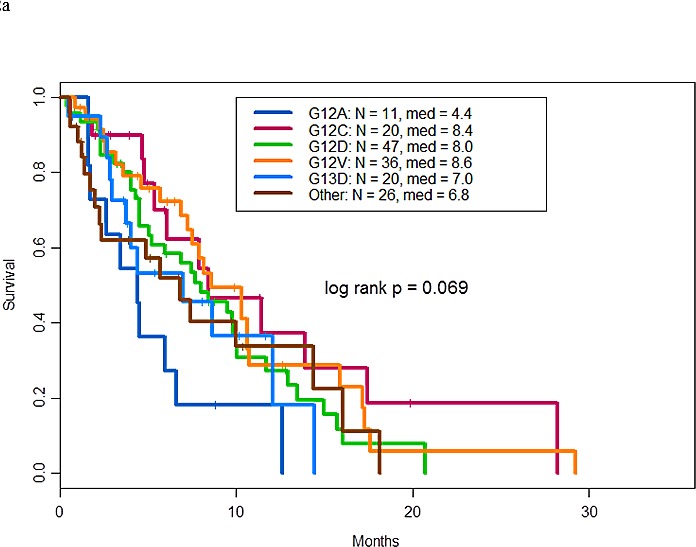

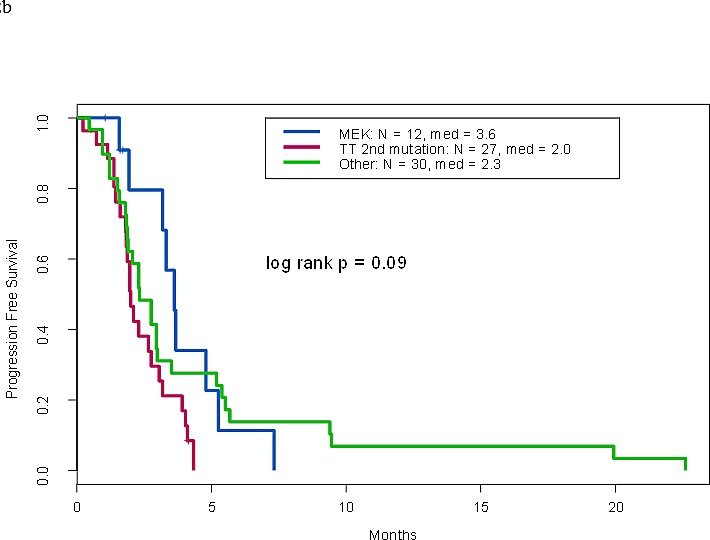

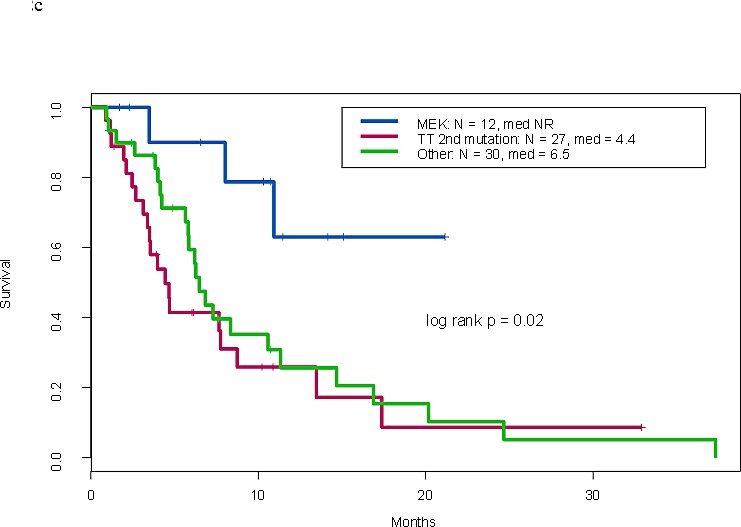

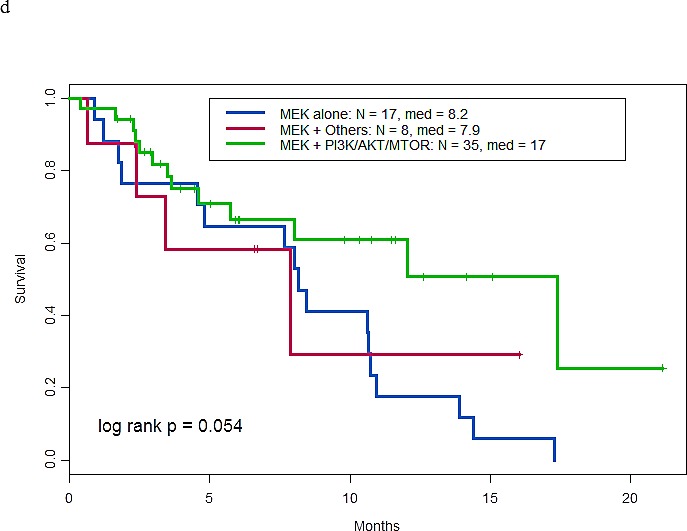
**a.** Overall survival by *KRAS* mutation types. **b.** Progression free survival of patients with *KRAS* and other molecular aberrations by treatment type. **c.** Overall survival of patients with *KRAS* and other molecular aberrations by treatment type. **d.** Overall survival of patients treated with MEK inhibitor containing trials by type of therapy

Best response by RECIST, PFS, and survival by specific *KRAS* mutation, tumor histology, and type of therapy are shown in Table 4. The clinical outcomes varied with the specific mutation (for instance, patients with the G12A mutation appeared to have poorer outcomes than those with other mutations), but the differences were not statistically significant (p=0.07) (Figure 2a).

**Table 4:** Clinical outcomes of patients with *KRAS* mutations

	Therapy	Evaluable	PR+SD≥6 Months (RECIST)	*P*	Median PFS, months	*P*	Median Survival, months	*P*
*KRAS* mutation				0.60		0.57		0.07
G12A	Any	10	0 (0%)		1.9		4.4	
G12C	Any	20	2 (10%)		2.6		8.4	
G12D	Any	44	7 (16%)		2.2		8.0	
G12V	Any	35	7 (20%)		2.3		8.6	
G13D	Any	20	3 (15%)		2.2		7.0	
Other	Any	23	2 (9%)		2.1		6.8	
Patients with *KRAS* mutation alone								
Colorectal	MEK-containing	12	1 (8%)	0.54	1.9	.94	5.7	0.06
	Other	73	4 (5%)		2.1		7.5	
Lung	MEK-containing	23	4 (17%)	1.00	3.2	.71	7.9	0.13
	Other	10	2 (20%)		3.2		17.6	
								
Pancreas	MEK-containing	5	2 (40%)	0.07	11.0	.08	14.4	0.27
	Other	12	0 (0%)		1.9		4.5	
Other tumors	MEK-containing	7	4 (57%)	0.34	7.7	.44	12.1	0.54
	Other	15	4 (27%)		2.2		9.8	
Total *KRAS* mutation alone	MEK-containing Other	47 110	11 (23%) 10 (9%)	0.02	2.8 2.1	.10	8.0 7.5	0.59
Patients with KRAS and other molecular alterations^§^				0.044		0.09		0.02
	MEK-containing	9	2 (22%)		3.6		Not reached	
	Targeted therapy matched the other alterations^¤^	24	0		2.0		4.4	
	Other, non-matched	33	5 (15%)		2.8		6.8	
Total KRAS^*^ mutation	MEK-containing vs. Other	56 167	13 (23%) 15 (9%)	0.005	3.3 2.2	0.09	8.4 7.0	0.38

§ The total number of patients with *KRAS* and other molecular alterations was 69 (66 were evaluable for response; all 69 patients were evaluable for PFS and OS).

¤ All the targeted therapy matching the other alterations were inhibitors of the PI3K/AKT/mTOR pathway.

* The total number of patients who received MEK was 60 (56 patients were evaluable for response; however, all 60 patients had data for PFS and OS). The total number of patients who received other therapy was 177 (167 had clinical benefit data; all 176 had data for PFS and OS).

Univariate and multivariate analyses of the prognostic significance of baseline characteristics of patients with *KRAS* mutations are summarized in Supplemental Table 1. Briefly, previously reported prognostic covariates (lactate dehydrogenase level, serum albumin level, number of metastatic sites, and ECOG performance status) for patients with advanced cancer treated on phase I clinical trials were confirmed in this analysis. Furthermore, the presence of additional mutations was found to be a good prognostic feature (HR: 0.7; p=0.03). Interestingly, after adjustment for other variables, G12V was found to be associated with longer survival compared to G12A (p=0.008; Supplemental Table 1).

### Outcomes of patients with KRAS mutations by therapy

The median number of phase I trials that patients with a *KRAS* mutation were treated on was one (range, 1-5). Clinical outcomes, including clinical benefit (partial response and stable disease ≥ 6 months per RECIST), PFS, and OS, are summarized in Table 4. Of 365 patients with *KRAS* mutations, 223 were evaluable for response. The other patients were excluded for the following reasons: loco-regional therapy (n=54), decline in performance status (n=28), consent withdrawal (n= 14), loss of follow-up (n=11), early toxicity (n=8), or other (n=27).

Of 157 patients with a *KRAS* mutation and no additional known alterations, 47 received a MEK inhibitor--containing therapy and 110 received non-MEK inhibitor therapy including another targeted agent, chemotherapy, or both. In subset analyses by tumor type, no significant difference was noted in outcomes between patients treated with a MEK inhibitor--containing therapy and those treated with other therapies. However, a trend towards a higher rate of clinical benefit was noted in patients with pancreatic cancer treated with MEK inhibitor--containing therapy compared to other therapies (p=0.07). When the total number of patients with a *KRAS* mutation was analyzed regardless of tumor type, the rate of clinical benefit of patients treated with a MEK inhibitor--containing therapy was higher than that of those treated with other therapies (p=0.02).

Of 66 patients with *KRAS* mutations and other molecular alterations, nine received a MEK inhibitor--containing therapy, 24 received therapies matched with other alterations (all targeted against alterations in the PI3K/AKT/mTOR pathway), and 33 received non-matched therapies. The overall clinical benefit rates were 22%, 0%, and 15%, respectively (p=0.04). In addition, patients who received a MEK inhibitor--containing therapy had longer PFS (p=0.09) and longer overall survival (p=0.02) compared to the other treatment groups (Figure 2b and 2c).

When all 223 patients with a *KRAS* mutation with or without an additional alteration were included in the analysis, MEK inhibitor-containing therapy had a statistically significantly higher rate of clinical benefit compared to other therapies (23% vs. 9%, respectively; p=0.005). A trend towards longer PFS was also noted in the MEK inhibitor group compared to others (p=0.09). No difference in overall survival was noted (p=0.38).

Of the 60 patients who received a MEK inhibitor--containing therapy, 35 also received a PI3K/AKT/mTOR pathway inhibitor, 17 received a MEK inhibitor only, and eight also received other targeted or cytotoxic therapy. Their respective median OS were 17, 8.2 and 7.9 months (p=0.054) (Figure 2d).

## DISCUSSION

To our knowledge, this is the first systematic analysis of the clinical outcomes of patients with *KRAS* mutations treated on phase I clinical trials. We found that *KRAS* mutations vary by tumor type. This heterogeneity in amino acid substitution is thought to be associated with differences in the clinical course of the disease. The use of MEK inhibitors in patients with *KRAS-*mutated advanced cancer was associated with higher clinical benefit rates compared to other therapies.

In our series, the proportion of tested patients with pancreatic cancer with *KRAS* mutation was similar to that reported in the COSMIC database[20]. However, the proportions of tested patients with colon and lung cancers with *KRAS* mutation were slightly higher than published data, perhaps in part reflecting the referral pattern in our Phase I Program, which may be affected by the availability of MEK inhibitor trials. This discrepancy can also be explained by differences in the detection method, histology subtypes, disease stage, and other factors.

Our analysis showed that *KRAS* mutation by specific amino acid substitution varies by tumor type. The specific *KRAS* mutations in colorectal cancer were similar to those previously reported in a smaller series, in which G12D and G12V were the two most commonly seen mutations [21]. Furthermore, the various specific *KRAS* mutations seen in lung cancer in our patient group were similar to those previously reported [22].

One of the intriguing findings of our analysis was the association of the *KRAS* G12V mutation with longer survival compared with the *KRAS* G12A mutation (p=0.008; Supplemental Table 1). This appears to be in line with preclinical and clinical studies demonstrating that specific *KRAS* mutations are associated with heterogeneous outcomes [22-24].

In our analysis, the use of MEK inhibitors was associated with an improved clinical benefit rate compared with other therapies in patients with a *KRAS* mutation (23% vs. 9%; p=0.005) (Table 4). This better disease control with MEK inhibitor-containing trials translated into a trend toward longer PFS in the MEK inhibitor group compared with others (3.3 vs. 2.2 months; p=0.09). However, no statistical difference in overall survival was noted between the two groups (8.4 vs. 7 months, p=0.38), perhaps because patients were treated with subsequent therapies. In addition to the relatively small number of patients, the short PFS seen in our analysis may be explained by the poor-prognosis patient population, the development of resistance to MEK inhibitors, or the development of other driver alterations by the time the patients started on treatment (archival tissue was used for molecular profiling in most patients). Our results are compatible with emerging data demonstrating that MEK inhibitors have promising antitumor activity in patients with *KRAS*-mutated cancer[18].

We also found that the presence of other molecular alterations in addition to *KRAS* alterations was associated with longer survival (Supplemental Table 1). The reason is unknown, but the numbers of patients with a KRAS mutation and 1 or ≥2 additional molecular alterations were too small compared to patients with a sole KRAS mutation (72, 37, and 256 patients, respectively) to draw meaningful conclusions. Clinical studies should explore the clinical significance of additional alterations in patients with tumor *KRAS* mutations. For instance, additional alterations may partially negate the effects of the *KRAS* mutation.

These results are hypothesis-generating, since they were derived from a retrospective analysis and not from a randomized trial. Therefore, one cannot distinguish between the prognostic value and the predictive value of certain types of KRAS mutations.

Our data also demonstrated that patients with *KRAS* mutations had a higher proportion of *PI3KCA* alterations than patients with wild-type *KRAS* (24% vs. 10%, respectively; p<0.0001) and that patients with wild-type KRAS had a higher proportion of BRAF mutations than patients with KRAS mutations (7% vs. 0.4%, p=0.0002) (Table 3). The coexistence of PI3K and KRAS mutations and the mutual exclusion of KRAS and BRAF mutations had been previously reported in a subset of patients with colorectal cancer [25, 26]. Furthermore, *KRAS* and *EGFR* mutations coexisted in 4 patients (2 patients with pancreatic cancer, 1 with CRC and 1 with NSCLC). Various reports showed that *KRAS* and *EGFR* mutations are mutually exclusive in patients with lung cancer[27-30]; however, some cases were previously reported to harbor both EGFR and KRAS mutations[31-33].

In both preclinical and clinical settings, the co-existence of both *KRAS* and *PI3KCA* alterations was reported to be a negative predictor of response to *PI3K* inhibitors[26, 34-37] and MEK inhibitors[38]. Both PI3K/AKT/mTOR and RAS/RAF/MEK/ERK are downstream pathways of the KRAS protein[39, 40]. Our data showed that treatment with a MEK inhibitor resulted in better outcomes than treatment with PI3K pathway inhibitors. Other investigators have demonstrated that tumors in xenograft models with both *PI3K* and *KRAS* molecular alterations regressed upon MEK inhibition and had a less-pronounced response to PI3K inhibition[41], perhaps suggesting that *KRAS* mutation may represent the driver mutation. Whether the combination of MEK and PI3K inhibitors is associated with better outcomes than either inhibitor alone is currently being investigated in clinical trials.

Our analysis has several limitations, which are typical for retrospective analyses for patients with any tumor type treated with targeted therapy based on genetic alterations. These limitations include relatively small numbers of patients with specific targetable alterations per tumor type, not including alterations other than KRAS mutations that can activate the MAPK pathway (as such alterations were not tested routinely in CLIA-certified laboratories), and various dose levels and various drugs used in the analyses, resulting in large tested hypotheses that cannot rule out false positive conclusions.

Prospective studies assessing the prognostic significance of various *KRAS* mutations will help elucidate the role of KRAS in human carcinogenesis. The development of novel agents for treating patients with KRAS mutations and therapeutic strategies that include novel agents combined with other targeted therapies, chemotherapy and/or immunotherapy is urgently needed. In this direction, the National Cancer Institute is launching a large-scale project to develop therapeutic strategies against cancers driven by *RAS* with the contribution of national scientific leaders and using core facilities and modern technology.

## METHODS

### Patients

We identified 1,781 consecutive patients with advanced cancer who were referred to the Phase I Clinical Trials Program at The University of Texas MD Anderson Cancer between November 2006 and March 2013 and who had undergone tumor molecular analysis for *KRAS* mutations as part of molecular profiling. Patients referred to the Phase I Clinic were of various ages, had advanced/metastatic cancer that was refractory to standard therapy or had relapsed after standard therapy, or had a tumor for which there was no standard therapy available. All protocols in the Phase I Clinical Trials Program required that participants have evidence of evaluable or measurable disease according to Response Evaluation Criteria in Solid Tumors (RECIST) guidelines[42, 43] and an Eastern Cooperative Oncology Group (ECOG) performance status of 0-2. Additional eligibility criteria varied according to the protocol on which the patient was enrolled. All patients provided written informed consent prior to enrollment onto a trial. All trials, as well as this analysis, were performed with the approval of and in accordance with the guidelines of the MD Anderson Cancer Center Institutional Review Board.

### Analysis of molecular alterations

Molecular profiling had been performed in a Clinical Laboratory Improvement Amendments (CLIA)-certified Molecular Diagnostics Laboratory, as previously described[44] or at Foundation Medicine, Inc.

### Therapy

Treatment was selected as previously described[44]. Briefly, patients whose tumors had a molecular aberration were preferably treated on a clinical trial with a matched targeted agent, when available. The allocation of patients to investigational treatment varied according to protocol availability, eligibility criteria, histologic diagnosis, the patient's prior response to therapy, potential toxicity, insurance coverage, and patient preference or physician choice. Patients treated with regional therapy were excluded from the outcome analysis.

The patients with tumors harboring *KRAS* mutations were treated with MEK inhibitor-containing therapy when a clinical trial was available. Patients were treated in the following clinical trials if the eligibility criteria were met: NCT01138085, NCT00454090, NCT01378377, NCT00687622, NCT01155453, and NCT01337765. If ≥ 2 molecular alterations were present, patients were treated in a matched trial that targeted both alterations, if available.

The types of treatment were categorized as (a) MEK inhibitor--containing therapy or (b) other therapies (targeted therapy with or without cytotoxic agents). If an additional alteration to the *KRAS* mutation was present, the treatment was categorized as follows: (a) MEK inhibitor--containing therapy, (b) targeted therapy for the additional alteration, when applicable, or (c) other therapies.

### Endpoints and statistical methods

Best response was assessed using imaging studies performed every two cycles (1 cycle = 3-4 weeks, depending on the protocol) by an MD Anderson radiologist. Tumor measurements were confirmed independently by a physician in the response assessment clinic within our department using RECIST guidelines applicable at the time of the patient's response assessment. Clinical benefit (CB) from any therapy was defined as complete response (CR) or partial response (PR) or stable disease (SD) for ≥ 6 months. Progression-free survival (PFS) was measured from the first day of treatment on a clinical trial until the patient came off study because of disease progression or death, whichever came first. Overall survival (OS) was measured from initiation of treatment on a clinical trial until death from any cause or last follow-up.

Patients' characteristics at the time of presentation in the Phase I Clinic were analyzed using descriptive statistics. Categorical data were described using contingency tables, including counts and percentages. Continuously scaled measures were summarized by median and range. The association between two categorical variables was examined using the chi-squared test. Survival and PFS were estimated using the Kaplan-Meier method, and survival was compared between groups using the two-sided log-rank test. Survival and PFS were analyzed based on the specific type of *KRAS* mutation and the type of treatment. Statistical analyses were carried out using TIBCO Spotfire S+ 8.2 for Windows (TIBCO Software, Inc.).

## SUPPLEMENTARY MATERIAL AND TABLE


