# Supplementary material for: Outcomes of patients with advanced cancer and KRAS mutations in phase I clinical trials
Source: Oncotarget. 2014 Aug 10;5(19):8937–46. doi: 10.18632/oncotarget.2339 (PMC4253408; doi:10.18632/oncotarget.2339)
Supplement: Supplementary file 1 [file oncotarget-05-8937-s001.pdf]

Outcomes of patients with advanced cancer and *KRAS* mutations in phase I clinical trials

Supplementary Material

Supplemental Table 1: Survival analysis of patients with *KRAS* mutations

Phase I Prognostic Characteristics	Univariate Analysis			Multivariate Analysis	
	**Patients, n**	**Median OS**	**p-value**	**HR (95%)**	**p-value**
**Number of prior therapies**			0.48		
0-3	191	8 (7, 11)		1.0	
>3	174	8 (7, 10)		1.2 (0.9, 1.6)	0.17
**Number of metastatic sites**			**<0.0001**		
**≤2**	233	10 (8, 13)		1.0	
**>2**	132	7 (5, 8)		1.6 (1.2, 2.1)	**0.001**
**ECOG-PS**			**<0.0001**		
0	109	12 (11,15)		1.0	
1	219	7 (6, 8)		1.5 (1.1, 2.1)	**0.006**
2,3	33	3 (2, 7)		3.2 (2.0, 5.2)	**<0.0001**
**Albumin**			**<0.0001**		
<3.5	74	5 (4, 6)		1.0	
≥3.5	285	10 (8, 12)		0.5 (0.4, 0.7)	**<0.0001**
**LDH**			**<0.0001**		
≤618	200	12 (10, 15)		1.0	
>618	160	6 (5, 7)		2.0 (1.5, 2.6)	**<0.0001**
**Platelets**			**0.07**		
≤ 400,000	346	8 (7, 10)		1.0	
> 400,000	17	5 (3, NR)		1.7 (0.9, 3.1)	0.09
**Gastrointestinal tumors**			**0.006**		
No	103	12 (8, 15)		1.0	
Yes	269	8 (7, 9)		1.2 (0.8, 1.6)	0.33
**Number of associated molecular alterations**			**0.01**		
0	256	8 (7, 9)		1.0	
1	72	8 (6, 13)		0.7 (0.5, 1.0)	**0.03**
≥2	37	17(11, NR)			
**Mutation type**			0.42		
G12A	25	5 (4, 16)		1.0	
G12C	41	8 (6, 16)		0.6 (0.3, 1.1)	**0.08**
G12D	106	8 (6, 10)		0.7 (0.4, 1.2)	0.22
G12V	80	9 (8, 11)		0.5 (0.3, 0.8)	**0.008**
G13D	40	7 (4, 28)		0.6 (0.3, 1.1)	0.11
Other	64	10 (7, 15)		0.6 (0.3, 1.0)	**0.04**

LDH: lactate dehydrogenase, ECOG: eastern cooperative oncology group, OS: overall survival
